# Development of a formula for scoring competence of bovine embryos to sustain pregnancy

**DOI:** 10.1016/j.bbrep.2024.101772

**Published:** 2024-07-02

**Authors:** Maria Belen Rabaglino, Peter J. Hansen

**Affiliations:** a Department of Population Health Science, Faculty of Veterinary Medicine, Utrecht University, 3584 CL, Utrecht, the Netherlands; b Dept. of Animal Sciences, University of Florida, Gainesville, FL, USA

## Abstract

Embryo transfer in cattle and other species is a key reproductive technology to improve genetic merit. However, pregnancy loss after embryo transfer is still a major barrier to optimal utilization of the technology. Furthermore, the lack of a method to objectively quantify embryonic competence hinders investigations aimed at improving the competence of an embryo. Based on the knowledge that bovine embryos have an inherent molecular signature that determines their ability for pregnancy establishment which can result in distinct gene expression profiles, we have previously integrated transcriptomic data from independent experiments to identify eight genes capable of predicting embryo competence for survival with high accuracy. In this study, we developed a function for the R software containing a mathematical formula based on the model coefficients to yield an embryonic competence index (ECI) according to the expression of those eight critical genes. Application of the function to a gene expression dataset generates a quantitative ECI value for each embryo that can be employed in statistical analyses when performing an experiment. The folder with the R project and required datasets can be found in https://zenodo.org/records/12515587.

## Highlights

• In cattle, pregnancy loss after embryo transfer diminishes reproductive efficiency.
• There is no method to objectively quantify the embryo ability to sustain pregnancy.
• Previously, we identified eight biomarker genes that predicted embryo competence.
• Based on these genes, we developed a formula to calculate embryo competence index.
• Here, we introduce an R function that yields the index in an expression dataset.

## 1 Introduction

Technological advances have transformed production of animal-based foods thanks to enormous achievements in enhancing the genetic gain in breeding populations and in managing the environment in which animals express their genetic potential. One technique that shows great promise for continued improvements in genetic gain and for managing reproductive function in cattle is the in vitro production of embryos. These embryos are an important tool for enhancing genetic selection on the female side [1], can themself be genotyped for genetic selection before transfer [2], and can be used as a management tool to improve fertility in certain cases [3] or to produce offspring of a different genotype than the birth mother [4]. Moreover, it is often more inexpensive to produce embryos in vitro than in vivo, genetic material can be obtained from very young or even pregnant females, and oocytes (female gametes) from animals sent to the food chain can be recycled. For these and other reasons, the transfer of in vitro produced (IVP) embryos has been rapidly expanding. In 2022, the number of transferred IVP embryos was 1,189,699 as compared to 368,783 for embryos produced in vivo [5].

However, a limitation of the embryo transfer technology is the lack of a highly accurate method for selection of embryos with high competence to establish pregnancy. The most widely used method still relies on subjective morphology assessments via light microscopy following the guidelines of the International Embryo Technology Society that lacks accuracy and repeatability [3]. Furthermore, the optimal effectiveness of the in vitro production of embryos technology for cattle production is hindered by the reduced ability of an IVP embryo to establish a sustained pregnancy after transfer compared to an embryo produced in vivo. Much effort has been made worldwide to investigate the several complex aspects of in vitro production involved from the in vitro fertilization of the oocytes to pregnancy establishment [6]. Progress in improving pregnancy success after transfer is limited by the demands of statistical power analysis: hundreds of transfers per treatment are required to reliably detect treatment effects on pregnancy outcomes [6]. Therefore, what is needed are reliable biomarkers of an embryo's competence to establish pregnancy so that experiments using the markers can be performed to objectively identify changes in in vitro procedures that are most likely to increase embryo survival after transfer.

It has been repeatedly demonstrated that embryos capable of establishing and sustaining a pregnancy have a different transcriptome than those that do not survive [[7], [8], [9], [10]]. Based on this concept, we applied machine learning methods to integrated molecular embryo data to further refine biomarker genes for pregnancy success [11]. A series of bioinformatic steps were performed to identify candidate genes that best discriminated between demi-blastocysts produced in vivo that resulted in a pregnancy at day 60 of gestation (PR) vs those that did not (NP). From a set of 1040 biologically relevant genes, there were eight genes that caused full discrimination between the PR and NP samples. Of the eight biomarker genes, three were more expressed in PR embryos; glutathione S-transferase omega 1 (*GSTO1*), chondroitin sulfate synthase 1 (*CHSY1*) and triosephosphate isomerase 1 (*TPI1*); while the other five; tyrosine 3-monooxygenase/tryptophan 5-monooxygenase activation protein gamma (*YWHAG*), cyclin A2 (*CCNA2*), LSM4 homolog, U6 small nuclear RNA and mRNA degradation associated (*LSM4*), cyclin-dependent kinase 7 (*CDK7*) and eukaryotic translation initiation factor 4A3 (*EIF4A3*), were increased in expression in NP embryos.

The embryo competence model built with these eight biomarker genes was validated through Bayesian logistic regression or neural networks, training the model with their expression in the in vivo produced PR and NP blastocysts [11]. Embryo developmental fate was predicted in four external datasets consisting of IVP blastocysts (i) competent or not, or (ii) exposed or not to detrimental conditions during culture, and elongated conceptuses (iii) of different length, or (iv) developed in the uteri of high or sub-fertile heifers. Predictions for each dataset were more than 85 % accurate, suggesting that these genes play a key role in embryo development and pregnancy establishment.

Data from each of these testing datasets were normalized to the training dataset through an add-on batch effect adjustment [12]; i.e., the test data were adjusted without changing the training data, to maintain the prediction rule every time that test and training data were normalized together. In other words, this approach requires to reduce the systematic differences in gene expression data between the query dataset and the dataset integrated from two different studies [7,10]. Therefore, the objective of the study described in the current report was to develop a function for the R software to wrap the steps needed to estimate the embryonic competence index (ECI) in a query dataset.

## 2 Materials & methods

Briefly, the steps that output an ECI in a query dataset consist of filtering the query data to retain the biomarker genes, normalize the filtered data with the training dataset through an add-on batch effect adjustment, and apply a regression formula to quantify the degree of embryonic competence for survival after transfer according to the expression of the eight biomarker genes for survival.

For the mathematical formula, we fitted a Bayesian logistic regression model to determine the coefficients of the equation by applying the train function of the caret package for R [13] and using “bayesglm” as a method. The final formula (R^2^ = 0.96) is as follow: ECI = 57.761 + 1.528(*GSTO1*) + 1.115 (*CHSY1*) + 1.803(*TPI1*) + −3.284(*YWHAG*) + −2.357(*CCNA2*) + −2.384(*LSM4*) + −1.418(*CDK7*) + −1.881(*EIF4A3*), where *GSTO1*, *CHSY1*, etc. are gene expression values normalized with the training dataset. A high value for the sum of the coefficients (i.e., high ECI) indicates high embryo competence while a low value would indicate low competence for embryo survival. To apply the function, the user should input a gene expression dataset (from RNAseq or microarrays technologies, or qRT-PCR) in a tabular format, i.e., the first column containing the transcript IDs (which can be ENSEMBLE gene or transcript, Entrez, Official Symbol, or RefSeq mRNA accession; Table 1), and the expression values for each sample in the remainder of the columns. In the case of qRT-PCR data, the relative expression values can be calculated as two raised to the dCT between the expression of the housekeeping gene(s) and each biomarker gene (i.e., 2 ^Ct_housekeeping−Ct_biomarker^).

**Table 1:** Transcript IDs and coefficient values for the eight biomarker genes whose expression is used in the formula to calculate the embryo competence index.

Gene Name	Official Symbol	Entrez	ENSEMBL gene	ENSEMBL transcript	RefSeq	Formula Coefficient
glutathione S-transferase omega 1	GSTO1	505642	ENSBTAG00000003989	ENSBTAT00000006245	NM_001075214.2	1.528
chondroitin sulfate synthase 1	CHSY1	281690	ENSBTAG00000007357	ENSBTAT00000009678	NM_001191157.1	1.115
triosephosphate isomerase 1	TPI1	281543	ENSBTAG00000019782	ENSBTAT00000026358	NM_001013589.3	1.803
tyrosine 3-monooxygenase/tryptophan 5-monooxygenase activation protein gamma	YWHAG	286862	ENSBTAG00000004077	ENSBTAT00000005327	NM_174793.2	−3.284
cyclin A2	CCNA2	281667	ENSBTAG00000004943	ENSBTAT00000006503	NM_001075123.1	−2.357
LSM4 homolog, U6 small nuclear RNA and mRNA degradation associated	LSM4	613634	ENSBTAG00000008578	ENSBTAT00000011312	NM_001035436.2	−2.384
cyclin-dependent kinase 7	CDK7	515462	ENSBTAG00000011046	ENSBTAT00000014667	NM_001075715.1	−1.418
eukaryotic translation initiation factor 4A3	EIF4A3	515145	ENSBTAG00000016023	ENSBTAT00000021327	NM_001046188.2	−1.881

The folder can be downloaded from: https://zenodo.org/records/12515587. After extracting the files in a known directory, and assuming that R/R studio are installed, the user can follow the instructions in the README file or in the step-by-step pdf file included in the folder. Briefly, the user should click on the ECI_project.Rproj file, which will open Rstudio, and access the function by clicking on the “FormulaECI.R” file, in the Files tab. The formula requires the bapred package [12]. After running the formula, the required datasets should be loaded into the environment. The users can add their own dataset to the “query_datasets” folder and change the name of the example dataset (“GSE130954_BlastoIVT_PR.txt”) to the corresponding name of the input dataset. This file should be in a table-like structure with transcript IDs in rows and samples IDs in columns. The “query_datasets” folder contains several files as examples. After loading the dataset of interest into the R environment, the function can be applied by running “embryo_index(data)”. The function will output a table with the calculated ECI for each sample in the query dataset. A word of caution is that the formula requires the expression of the eight biomarker genes to calculate the ECI. Therefore, if any sample has a zero value in any of these genes, the function will generate a warning and the sample will be removed.

## 3 Results & discussion

As example, we applied the function to gene expression data from datasets that we had employed in our initial study to validate the predictive model [11] and to datasets downloaded from the Gene Expression Omnibus database. These datasets correspond to: (i) in vitro and in vivo produced demi-blastocysts where one half was used for gene expression and the other half transferred to cows to determine pregnancy outcome (NP = 33; PR = 20) [7,8], (ii) cloned and in vitro fertilized 2-cell embryos (n = 6) and blastocysts (n = 6) [14], (iii) trophoblast of in vitro Day 14 embryos that were exposed to vitrification (n = 7) or not (n = 5) during the blastocyst stage [15], (iv) in vitro Day 14.5 embryos that were supplemented (n = 10) or not (n = 9) with interleukin 6 (IL6) on day 5 post-fertilization [16], and (v) short (n = 17) and long (n = 9) Day 17 conceptuses developed in the uteri of high or sub-fertile heifers [17]. For each study, the effect of each variable(s) and their potential interaction on the resulting ECI were compared by ANOVA using the GLM procedure for SAS Studio (Release 3.81).

**Fig. 1:**
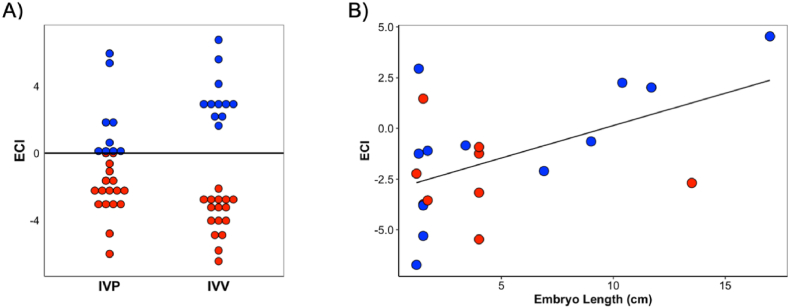
Determination of embryonic competence indexes (ECI) for (A) demiembryos produced in vitro (IVP) or in vivo (IVV) that resulted in pregnancy (blue dots) or not (red dots) and (B) elongated day 17 conceptuses recovered from high fertile (blue dots) or subfertile (red dots) heifers. (For interpretation of the references to colour in this figure legend, the reader is referred to the Web version of this article.)

For each corresponding dataset, several conditions demonstrated higher average ECI when compared to the other condition. For dataset i, demi-embryos that resulted in pregnancy had higher ECI vs those that did not (p < 0.0001) for both in vitro (1.79 ± 2.31 vs −2.28 ± 1.54) and in vivo produced embryos (3.39 ± 1.54 vs −3.70 ± 1.26; Fig. 1A). For dataset ii, blastocysts had higher ECI than 2-cell embryos, regardless of whether they were produced in vitro or after somatic cell nuclear transfer (2.53 ± 1.28 vs −3.62 ± 0.59, p < 0.0001). Compared side by side, a blastocyst is expected to be more competent than a 2-cell embryo, which has not yet undergone embryonic genome activation and, therefore, still expresses the oocyte's transcriptome [18]. For dataset iii, Day 14 embryos transferred fresh at the blastocyst stage had higher ECI vs those transferred after vitrification (0.35 ± 1.03 vs −1.46 ± 1.03, p = 0.01). The authors of this study reported that Day 14 embryos that underwent vitrification at the blastocyst stage showed rapid cell proliferation, which could increase the risk of DNA damage. Furthermore, genes involved in energy metabolism, which play a critical role in embryo survival [19], were de-regulated on these embryos, probably because of a negative energy balance [15]. Thus, Day 14 embryos that were transferred after vitrification should have been less competent, or less able to sustain a pregnancy, than those that were transferred fresh, supporting the ECI results. For dataset iv, Day 14 embryos supplemented with IL6 during culture had higher ECI than those that were not treated (−0.04 ± 2.29 vs −1.98 ± 1.22, p = 0.03). There were no differences in the ECI between ovoid or tubular embryos. However, the ECI values between IL6 treated or not treated embryos were more different for ovoid embryos (0.39 ± 2.5 vs −2.09 ± 1.06) than tubular embryos (−0.7 ± 2.09 vs −1.85 ± 1.57). The authors of this study found 1067 differentially expressed genes (DEG; FDR<0.05) between IL6 and control embryos. The number of DEG for this comparison was 228 when only ovoid embryos were considered, but only eight DEG if the comparison was done with the tubular embryos. Furthermore, there were only two DEG between tubular and ovoid embryos [16]. Interleukin-6, an anti-inflammatory cytokine, is a potential embryokine: pregnancy loss for embryos produced in culture medium with IL6 was lower than pregnancy loss for embryos cultured without IL6 (7 % vs 33 %) [20]. The transcriptome of Day 14 embryos supplemented with IL6 suggested a favourable embryonic and placental development and improved survival. Thus, the ECI values support the main findings of this study, i.e., IL6 treatment favoured embryonic development, and the effect was more evident in ovoid embryos than in tubular ones. Finally, for dataset v, long embryos had higher ECI vs short embryos at Day 17 (1.75 ± 2.98 vs −2.27 ± 2.44, p = 0.004). Furthermore, the length of the embryo was positively correlated with the ECI (Fig. 1B; R2 = 0.27, p = 0.01), reinforcing the finding that short conceptuses fail to upregulate several interferon-dependent and independent genes in the endometrium [21] and they are likely less competent than long conceptuses [19].

In summary, the R function introduced here can be used for research purposes to measure the effect of different treatments on the embryo ability to sustain a pregnancy and generate a quantitative value for each embryo that can be employed in statistical analysis. The limitations rely on the fact that the formula is based on the expression of only eight genes, and variations in expression due to technical factors in even one gene can impact the ECI value. Therefore, the researcher should consider this limitation if one or a few samples yield extreme ECI. Further research will aim to improve the formula to account for technical variations by analysing the expression distribution across all the queried samples. In the future, this study can establish the basis for the development of a highly accurate method for the objective evaluation and selection of IVP and in vivo produced bovine embryos before transfer into the surrogate uterus in order to maximise pregnancy, which could strongly and positively impact the cattle industry.

## Funding

This research did not receive any specific grant from funding agencies in the public, commercial, or not-for-profit sectors.

## CRediT authorship contribution statement

**Maria Belen Rabaglino:** Writing – review & editing, Writing – original draft, Visualization, Validation, Software, Methodology, Formal analysis, Data curation, Conceptualization. **Peter J. Hansen:** Writing – review & editing, Validation, Resources, Methodology, Conceptualization.

## Declaration of competing interest

The authors declare that they have no known competing financial interests or personal relationships that could have appeared to influence the work reported in this paper.
